# Peppering versus Single Injection Technique in Tennis Elbow - A Prospective Comparative Study

**DOI:** 10.5704/MOJ.2203.013

**Published:** 2022-03

**Authors:** YR Prakash, A Dhanda, KL Yallapur, SS Inamdar, GT Darshan, M Ramakrishna

**Affiliations:** 1 Department of Orthopaedics, Bangalore Medical College and Research Institute, Bangalore, India; 2 Department of Orthopaedics, Vijayanagar Institute of Medical Sciences, Ballari, India

**Keywords:** single injection technique, peppered injection technique, PRTEE, lateral epicondylitis

## Abstract

**Introduction:**

Lateral epicondylitis is a common condition causing severe incapacitating pain. Several methods of treatment have been approached for its management. In our study we aim to compare the results of injecting steroid and lignocaine mixture via single injection and peppered injection technique and analyse the outcome in each category.

**Materials and methods:**

A prospective randomised study comprising of 25 patients in each group (single vs peppered group) were included in the study after satisfying inclusion and exclusion criteria. Outcome of the treatment was measured in the form of Patient Related Tennis Elbow Evaluation (PRTEE) Questionnaire, Visual analogue score (VAS) and tenderness grading at two weeks, six weeks and six months after injection.

**Results:**

Results of our study showed that the mean PRTEE score was 22.36, 18.40 and 14.16 at 2 weeks, 6 weeks and 6 months following peppered injection as compared to 28.96, 21.84 and 25.32 in the single injection group (p value <0.05). VAS score at 2 weeks, 6 weeks and 6 months after the peppered injection was found to be 2.72, 1.72 and 1.36 and in the single injection group was 2.96, 1.92 and 2.72 at 2weeks, 6 weeks and 6 months, respectively (p value <0.05). On comparison of the 2 groups, there was a significant reduction of VAS scores at 6 months post-injection (p value <0.05) and PRTEE score at 6 weeks, 6 months in peppered injection group.

**Conclusion:**

The effects of peppered injection technique is seen to be advantageous over the single injection technique in the management of chronic lateral epicondylitis.

## Introduction

Lateral epicondylitis is a chronic condition characterised by pain at the common extensor origin over the lateral aspect of the elbow region. It was first described by Runge in the year 1873^1^, as commonly affecting women in their 5th and 6th decade. Although it may not necessarily be associated with playing tennis, it is seen in persons who perform repetitious movements involving the forearm, with elbow in extension^2^; like carpenters, musicians, or computer programmers. Prevalence of this condition is approximately 1-3% in general population^3-5^. Tennis elbow is usually regarded as a minor ailment, but it causes a nagging pain in the elbow region interfering with the day-to-day activities of the affected individuals, and in some instances, may flare up severely needing immediate intervention. Mechanical overload and repetitive stress leads to tendinosis and microtrauma at the extensor carpi radialis brevis muscle origin. This gradually progresses to a partial injury, which may lead to a full thickness tendon tear in untreated individuals^6^.

There are several treatment options available for lateral epicondylitis, conservative treatment in the form of oral analgesics and anti-inflammatory drugs, physiotherapy, application of tennis elbow braces and interventional procedures like intralesional injection of steroids, platelet rich plasma injection and arthroscopic or open surgery^7-8^. Intralesional injection of steroids have been in use in the treatment of tennis elbow since 1950^9^. Steroid injections relieve pain, reduce inflammation and improve mobility^10^. The significant reduction in pain provided by local steroid injection is however short lived and is seen to last for only about six weeks, but they have been found to be superior to oral analgesics and inflammatory drugs^11^. In a systematic review of randomised controlled trials conducted by Smidt *et al*, they concluded that corticosteroids appeared to be effective in the short term up to six weeks, although the optimal timing, the dosage and technique of injection needs to be further researched upon^12^. Tonks *et al* in their study advocated that steroid injection alone is the first line of treatment in patients with lateral epicondylitis requiring early return to daily activities^13^. There are two well-known techniques available for injection of intralesional steroid namely, single injection technique and peppering technique. Pruce *et al*^14^ first introduced the peppering technique in 1964. In this technique, after insertion of the needle, it is withdrawn, redirected and reinserted multiple times, without emerging out from the skin, which results in the formation of a hematoma which helps in faster healing of the degenerated tendon. This injection technique has also been utilised in delivering drug mixtures for lateral epicondyltis in several other studies such as those conducted by Altay *et al*^15^, Ghorpade *et al*^16^, Dogramaci *et al*^17^ and Kumar *et al*^18^. Our study is similar to the study conducted by Kumar *et al*^18^ with the aim to compare the outcomes of single injection versus peppering technique in the treatment of lateral epicondylitis and to analyse the results in our population in order to obtain greater validity to the findings.

## Materials and Methods

After obtaining Institutional Ethics Committee clearance (No.VIMS/MED/STAFF/SYN/67/2018-19), a prospective study was conducted on 50 consecutive patients of lateral epicondylitis presenting to the Orthopaedic outpatient department of our hospital, during the period of March 2018 to February 2019. The diagnosis of lateral epicondylitis was established based on clinical examination with tenderness over lateral epicondyle, a positive Cozen’s test and Mill’s manoeuvre. Magnetic resonance imaging (MRI) was not used in the diagnosis of the condition. Patients with acute lateral epicondylitis, aged above 18 years and not responding to conservative therapy (Symptoms and limitation of activity persist despite adequate analgesia and physiotherapy) for more than 3 months were included in our study. Patients less than 18 years of age, patients with evidence of intra-articular pathology (osteochondritis dissecans, inflammatory arthritis etc), local skin diseases, entrapment neuropathy (radial tunnel syndrome), any infective/neoplastic pathology were excluded from the study.

Sample size and Method of Calculation: Sample size was calculated based on the formula;


$$n\;=\;2\;[Z_{\alpha }+Z_{\left(1-\beta \right)}]2\;{\left(\sigma \right)}^{2}\;/\;d^{2},$$

where n is sample size; Zα is Standard table value for 5% level of significance; Z(1-β) is standard table value for 80%; σ is the standard deviation; d is difference between the mean. Considering a dropout rate of 10% a final value of n was measured to be 24.2 and hence a sample size of 25 in each group was chosen.

All patients were explained about the procedure and a written informed consent was taken for the same. Patients were divided into two groups based on systematic random sampling method with 25 patients in each group. Group 1 received injection by single injection technique and group 2 received injection by peppering technique. Routine investigations were performed along with RA factor, ESR and CRP to rule out inflammatory and infective pathology. AP and Lateral radiographs of the elbow was obtained. All patients were assessed initially using Visual Analogue Scale (VAS) and Patient Related Tennis Elbow Evaluation (PRTEE) questionnaire.

**Fig. 1:**
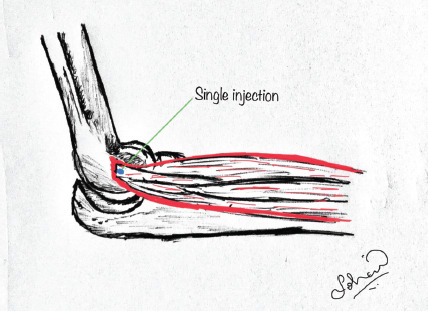
Single injection technique.

**Fig. 2:**
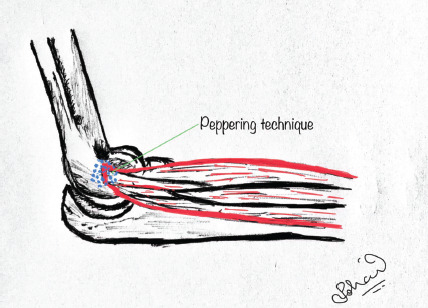
Peppered injection technique.

Patients were seated comfortably with the elbow in 90° flexion and forearm in pronation. The point of maximal tenderness is palpated, and the area is sterilised using 10% povidone-iodine solution. A mixture containing 1ml of triamcinolone acetonide and 2ml of 2% lignocaine solution is prepared and injected at the point of maximal tenderness using an 18-gauge needle. In the single injection technique, the skin is penetrated at the point of maximal tenderness, and the needle is inserted up to the bone, withdrawn a few millimetres and the drug mixture is deposited in this position entirely (Fig. 1). In the peppered injection technique, skin is penetrated at the point of maximal tenderness in a similar fashion; the needle is inserted up to the bone, withdrawn a few millimetres, a small quantity of the drug mixture is delivered here. This procedure is repeated several times in different directions without removing the needle completely from its initial point of entry in the skin (Fig. 2). Upon piercing the tendon sheath of the degenerated tendon, a cracking sensation is noted in most patients. The above process is stopped when the cracking sensation or the crepitation ceases. This procedure is usually not very painful as multiple punctures are made only on the tendon sheath and it causes no injury to the bone or periosteum^18^. Post-injection, sterile dressing was done and patients were started on oral anti-inflammatory drugs for three days. Strenuous activities were avoided in the affected limb. Physiotherapy in the form of wrist extension stretch, wrist flexion stretch, supination and pronation strengthening, and finger squeeze were advised after procedure in a gradual manner as tolerated by the patients. Patients were followed-up at two weeks, six weeks and six months post-injection using visual analogue pain score, PRTEE questionnaire and tenderness grading^19^.

## Results

The mean age of patients in the single injection group was 38.8 years with majority of the patients, 12 out of 25 (48%) between 31-40 years of age. Nine patients (36%) were between 41-50 years, 3 patients (12%) between 21-30 years and 1 patient (4%) between 51-60 years of age.

In the peppered injection group, the mean age was 38.48 years with majority of the patients; 13 out of 25(52%), between 31-40 years of age, 7 patients (28%) between 41-50 years, 3 patients (12%) between 21-30 years and 2 patients (8%) between 51-60 years of age.

The mean VAS score in the single injection group was 7.48 at the time of presentation which gradually started declining after injection to 2.96 at 2 weeks, and 1.92 at 6 weeks, which was statistically significant, but again started to increase at 6 months to 2.72. The mean VAS score in peppered injection group was 7.08 at the time of presentation which showed a steady progressive decline to 2.72, 1.72 and 1.36 at 2 weeks, 6 weeks, and 6 months, respectively. In comparison to the single injection group, the VAS score in the peppered injection group was lower even at six months. We noted a significant reduction (p value < 0.05) in the VAS scores in both the groups individually after injection at 2 weeks, 6 weeks as well as 6 months. VAS score comparison between the two groups revealed no significant difference at two weeks, and six weeks, however at six months post-injection we noted a significant difference between the two groups (Table I).

**Table I:** Comparison of VAS and PRTEE Scores between single and peppered injection groups

		Pre-Injection	2 Weeks	6 Weeks	6 Months
VAS Score	Single	7.48 ± 1.19	2.96 ± 0.84	1.92 ± 0.75	2.72 ± 0.85
	Peppered	7.08 ± 1.41	2.72 ± 0.74	1.72 ± 0.79	1.36 ± 0.48
	P Value	------	0.29	0.59	<0.05
PRTEE Score	Single	58.36 ± 7.72	28.96 ± 5.60	21.84 ± 4.11	25.32 ± 4.23
	Peppered	52.84 ± 5.81	22.36 ± 4.47	18.4 ± 3.47	14.16 ± 3.08
	P Value	------	3.34	<0.05	<0.05

The pre-injection mean PRTEE score in the single injection group was 58.36. The mean PRTEE score at 2 weeks, 6 weeks and 6 months after injection was noted to be 28.96, 21.84 and 25.32, respectively. Gradual decline in the PRTEE score was observed until six weeks of follow-up; however, at six months the PRTEE score was noted to be increasing. The pre-injection mean PRTEE score in the peppered injection group was 52.84. The mean PRTEE score at 2 weeks, 6 weeks and 6 months was noted to be 22.36, 18.4 and 14.16, respectively. Steady decline in the mean PRTEE scores were noted even at 6 months of follow-up as opposed to the findings in the single injection group. Significant reduction (p value < 0.05) was observed in the PRTEE score in both the single injection group as well as the peppered injection group following the injection at two weeks, six weeks, and six months (Table I). On comparison of the two groups, at two weeks post-injection, no significant difference was noted; however, it was noted that there was a significant reduction in the PRTEE score at 6 weeks and at 6 months with p value <0.05.

In the single injection group, the pre-injection assessment revealed that 56% of the patients showed Grade 3 and 44% of the patients had Grade 2 degree of tenderness^19^. In the peppered injection group, 48% of the patients showed Grade 3 tenderness prior to injection and 52% patients showed Grade 2 tenderness (Table II). Post-injection tenderness evaluated at 6 months after injection revealed that 32% patients in the single injection group had grade 2 tenderness, 52% patients had grade 1 tenderness and 16% patients had no tenderness. In the peppered injection group, 24% patients had grade 2 tenderness, 52% patients had grade 1 tenderness and 24% patients had no tenderness (Table II).

**Table II:** Comparison between pre-injection and post-injection tenderness among the two groups

Tenderness	Single injection group		Peppered technique group	
	Pre-injection	6 months post-injection	Pre-injection	6 months Post-injection
Grade 3	56% (14)	0	48% (12)	0
Grade 2	44% (11)	32% (8)	52% (13)	24% (6)
Grade 1	0	52% (13)	0	52% (13)
No Tenderness	0	16% (4)	0	24% (6)

## Discussion

Lateral epicondylitis is a debilitating condition for the patient, especially when present for a long duration of time. Multiple modalities of treatment have been attempted in the treatment of lateral epicondylitis as described in the literature^6^. Most of the patients report symptomatic improvement with non-operative measures within one year of treatment and only a small proportion of cases require surgical intervention. Many theories have been proposed in the etiology of tennis elbow, but the most widely accepted theory is that it is caused due to repeated trauma to the tendon of extensor carpi radialis brevis^4^.

The PRTEE questionnaire is a very reliable method of evaluating pain and disability in patients with chronic lateral epicondylitis. In a study conducted by Rompe *et al*20, it was noted that the PRTEE questionnaire was most sensitive to any change in the improvement or deterioration of patient’s symptoms following treatment, compared to other methods. Hence, we used PRTEE scoring to assess clinical outcome at each follow-up.

In our study, there was a significant reduction in the mean VAS score and mean PRTEE score at two weeks, six weeks and six months following injection in the peppered injection group which was comparable to a study conducted by Ghorpade *et al*16 as well as Dogramaci *et al*17. However, in the single injection group, the mean VAS score and the mean PRTEE score showed a significant reduction at two weeks and six weeks after injection and gradually seen to increase at six months. This finding was consistent with study conducted by Ghorpade *et al*16.

Pre-injection and post-injection tenderness was assessed in all patients in both groups, and it was noted that there was significant reduction in tenderness at six months post-injection in the peppered injection group as compared to the single injection group. These findings were consistent with the results seen with the study conducted by Kumar *et al*18.

The final results seen with our study is similar with those seen with the study conducted by Dogramaci *et al*17 who noted better results with injection by the peppering technique. It was also noted that the method of injection showed more significance in the outcome than the drug mixture. Okcu *et al*21 in his study observed that the long-term outcome of the treatment of lateral epicondylitis relies on the method of injection technique rather than the drug mixture being injected. He also concluded that the peppering technique is a more reliable and effective method of injection than the single injection technique. Altay *et al*15 in his study, used the peppering technique to compare local anaesthetic with a local anaesthetic-corticosteroid mixture and reported no significant difference between the two groups, further confirming the fact that the method of injection held more significance in the treatment of chronic lateral epicondylitis than the drug mixture itself.

Reddy *et al*22 in their study concluded that steroids and xylocaine are effective over a short-term basis, however PRP is effective on long term at 26-and 52-months follow-up as well. Varshney *et al*23 reported that single injection of PRP improves pain and function more than steroids in lateral epicondylitis and that these findings were sustained over a long time with no complications. Hsieh *et al*24 in their study reported no significant difference between injection of single dose of steroid versus single dose of lidocaine and found satisfactory functional outcomes with both the methods.

The strength of our study is that the research objectives were clearly stated, and explanations were provided for the measured outcomes. There were no complications noted in our study. Limitation of this study include the absence of MRI or Ultrasound to confirm the diagnosis and to assess healing of the tendon origin after injection, a short follow-up period, limited sample size and no proper randomisation.

The cracking and crepitus felt during the peppering technique is subjective leading to interobserver variability and thereby requires a uniform method of assessment of adequacy of the injection. Hence ultrasound guided injection can be adopted in further studies. We also recommend using MRI before and after injection to assess the amount of tendon degeneration and regeneration, respectively.

## Conclusion

Lateral epicondylitis is a chronic and debilitating illness and the fact that many modes of treatment exist shows that there is no one particular fool proof method. Our study concludes that the technique of injection seems to have a more important effect on the outcome than the drug mixture. The peppering technique for injection of the steroid/lignocaine mixture is a simple and effective technique in the relief of pain in chronic lateral epicondylitis and seems to have a better outcome as compared to the single injection technique.
